# Editorial

**Published:** 1854-01

**Authors:** 


### THE MEDICAL EXAMINER.

### PHILADELPHIA, JANUARY, 1854.

By the note of the former Editors in the December number, the readers of the Examiner were made aware of the change in its Editorial management.

In assuming the responsibility of Editorship of a Journal which has so extensive a circulation, and has always been so favorably received, we have the strongest incentive to exertion, to preserve for it the high character it has hitherto enjoyed.

We shall use every effort to make the "Record" a repertory of useful and scientific intelligence; sparing no pains to obtain for it, from every source that is open to us, all that is recent and instructive in medical literature.

Translations will be occasionally made, from the French and German, of such articles, as in our judgment will prove interesting to our readers.

In reviewing the books, &c. sent us, we shall strive to be honest and impartial; speaking the truth, uninfluenced by fear or favor, but we trust courteously, in every instance.

We must remind our readers, however, that the reputation of the Examiner depends in great measure upon its friends, as on them it relies for its most valuable department, its original communications; of which favors we respectfully solicit a continuance.

We gladly embrace the present opportunity to express our earnest wishes, that the gentlemen who have just retired from the Editorship of this Journal, will meet with that success as Professors to which their merits and abilities so justly entitle them.

